# Assessing the benefits of the “Intergalactic World” social emotional learning program for 8–12-year-old children in Portugal: perspectives from teachers and caregivers

**DOI:** 10.3389/fpsyg.2023.1233335

**Published:** 2023-08-28

**Authors:** Rita Antunes, Joana Alexandre, Maryse Guedes, Marisa G. Filipe, Manuela Veríssimo

**Affiliations:** ^1^Center for Research in Psychology for Positive Development, Lusíada University of Lisbon, Lisbon, Portugal; ^2^CUF Descobertas Hospital, Lisbon, Portugal; ^3^William James Center for Research—ISPA, Instituto Universitário, Lisbon, Portugal; ^4^ISCTE, University Institute of Lisbon, CIS_ISCTE, Lisbon, Portugal; ^5^Center of Linguistics, School of Arts and Humanities, University of Lisbon, Lisbon, Portugal

**Keywords:** social–emotional learning programs, internalizing behaviors, externalizing behaviors, caregivers, teachers

## Abstract

**Introduction:**

“Intergalactic World” is a new social–emotional program designed to reduce psychopathological symptoms and improve social and emotional skills in children aged 8–12. This study aims to evaluate the program’s benefits from teachers’ and caregivers’ perspectives, focusing on internalizing and externalizing behaviors.

**Methods:**

The findings were obtained through self-reported measures using a pretest-posttest design with a follow-up period, but with no control group. One hundred fifty-four children (*M* age = 9.66, *SD* = 0.78) participated in this intervention study. Eleven teachers completed the Teacher’s Report Form (TRF) for these children, and 133 caregivers completed the Child Behavior Checklist (CBCL). Participants without caregivers’ reports were excluded from the analysis. Data were collected at three-time points: before the intervention (T1), immediately after (T2), and 6 months after the implementation of the program (T3).

**Results:**

Results (*n* = 133) showed an effect of time on the Internalization scores (at T3 for teachers and T2 and T3 for caregivers) with no gender effect and a decrease in the perception of externalizing behaviors with a gender effect: Boys were perceived as exhibiting more externalizing behaviors than girls. However, these behaviors significantly decrease at T3 for teachers and at T2 and T3 for caregivers.

**Discussion:**

Despite its limitations, this study highlights the benefits of employing social–emotional programs to help reduce children’s internalizing and externalizing behaviors. A multi-informant approach enables a comprehensive analysis and provides insights into the child’s significant contexts and interactions with adults.

## Introduction

Social and emotional skills positively impact learning and important life outcomes, promoting positive social behavior and reducing conduct problems (Kankaraš and Suarez-Alvarez, 2019; Chatterjee Singh and Duraiappah, 2020). These skills are particularly important for children with behavioral problems, encompassing either internalizing or externalizing behaviors. Externalizing behaviors include hyperactivity, attention problems, and conduct problems (e.g., opposition). On the other hand, internalizing behaviors typically consist of self-directed difficulties (Achenbach and Edelbrock, 1978), such as anhedonia and negative moods and emotions (Schuman-Olivier et al., 2020), which are associated with various depressive and anxiety disorders (Hansen and Jordan, 2020).

The literature shows that boys usually display more externalizing behaviors than girls, whereas girls tend to exhibit more internalizing behaviors (Eme, 2016; Gutman and McMaster, 2020; Lau et al., 2021). Children and young people with behavioral problems suffer from emotional and behavioral regulation changes, leading to frequent referrals to mental health services and substantial burdens for families and organizations (Scott et al., 2001).

Despite the existence of several conceptual frameworks (e.g., Chernyshenko et al., 2018), the Collaborative for Academic, Social, and Emotional Learning (2012) defines social–emotional learning (SEL) as “the processes through which children and adults acquire and effectively apply the knowledge, attitudes, and skills necessary to understand and manage emotions, set and achieve positive goals, feel and show empathy for others, establish and maintain positive relationships, and make responsible decisions” (p. 9).

Delivering evidence-based SEL programs worldwide is an important pathway to enhance social–emotional competencies. However, the efficacy and effectiveness of SEL programs have been mainly studied in Anglo-Saxon countries, with fewer studies conducted in Ibero-American regions (Fernández-Martín et al., 2021).

### “Intergalactic World” program

The “Intergalactic World” was developed in response to the need for SEL programs for children attending primary and secondary schools in Portugal (Cristóvão et al., 2017; Antunes et al., 2022; Antunes, in press), which is considered an Ibero-American region according to some definitions. This eight-session prevention program (*cf.* details in Table 1) aims to promote self-regulation, self-control, and attentional focus in children aged between 8 and 12 years old.

**Table 1:** “Intergalactic World” program: goals and session activities (Antunes, in press).

Session number	Goals	Dynamics (1) and exercise key contents (2)
1	Understand the concept of informed consent/assent Promoting social–emotional competencies Main SEL domains: Self-knowledge Self-management Social consciousness Interpersonal relationship	(1) - Welcome and provide information. - Introduce group leaders. - Relaxation activity. - “My Intergalactic Passport.” - “My Superpowers”: Discovering my inner Superhero. - “My Superpowers” (Super Adventure). (2) - Relaxation dynamics mediated by deep breathing exercises and music. - Reflection about personal characteristics, behaviors, and feelings associated. - Discover and share what you consider to be your potential.
2	Promoting social–emotional competencies Main SEL domains: Self-management Social consciousness Interpersonal relationship	(1) - “An Intergalactic Day.” - “School in the Galaxy of Behavior.” - “My superpowers” (Super Creativity). (2) - Reflection exercises and interpersonal sharing about their daily routines vs. ideal routines (at school and at home). Sharing of tastes and interests vs. mediated by graph-expressive expression activities (e.g., drawing and free writing). - Exercise of mime and reflection in small groups.
3	Promoting social–emotional competencies Main SEL domains: Self-knowledge Self-management Social consciousness Responsible decision making	(1) - “The Theater of Intergalactic Emotions.” - “The Intergalactic Mirror.” - “My superpowers” (Super Energy). (2) - Reflection on feelings and emotions mediated by images of the four basic emotions (happiness, sadness, fear, and anger) and by dramatization and emotional venting exercises. - Sharing in small groups and then in a large group: Exercises in groups of two. - Face to face, define which child is the “mirror,” who copies the movements, and which one defines the movements. Then ask them to copy the movements of each other, in a large group mediated by different music, rhythms, and speeds.
4	Promoting social–emotional competencies Main SEL domains: Self-knowledge Self-management Social consciousness Interpersonal relationship Responsible decision making	(1) - “Intergalactic relaxation.” - “An apple at the ‘Intergalactic world.’” - “My superpowers” (Super Attention). (2) - Reflection and relaxation exercises mediated by imagery and dramatization (e.g., Asking them to move around the room as if they were an astronaut on the Moon) and share emotions and feelings associated. - Emotional ventilation exercises based on the five senses (sight, hearing, smell, touch, and taste), e.g., explore an apple as if it were “the first time” and share the associated experiences.
5	Promoting social–emotional competencies Main SEL domains: Self-knowledge Self-management Social consciousness Interpersonal relationship Responsible decision making And attentional focus in particular	(1) - “Discover Intergalactic Objects.” - The Party in Space.” - “My Superpowers”: What superpower would you like to receive? Design it and give it a creative name! (2) - Attention games like “find lost objects” in the “galaxy of feelings and behaviors” mediated by images and memorized exercises. - Exercise in pairs to build a fun moment for a party (e.g., a dance, a game, and a theater) and at the end build the “best party ever” in a large group.
6	Promoting social–emotional competencies Main SEL domains: Self-knowledge Self-management Social consciousness Interpersonal relationship Responsible decision making And attentional focus in particular	(1) - The Intergalactic School. - A House on Mars. - “My Superpowers” (Super Ideas and Super Happiness). (2) - Reflection exercises about school and friendships mediated by role-plays, mimiques, or drawing “our super planet”—do the same about home and family. - In a large group, build “the best school and the best house ever” mediated by role-plays, drawings, mimes, dramatizations, and creative writing exercises.
7	Promoting social–emotional competencies Main SEL domains: Self-knowledge Self-management Social consciousness Interpersonal relationship Responsible decision making	(1) - “A Statue in the Intergalactic Galaxy.” - “A toast to the Intergalactic Union.” - “My Superpowers” (Super Strength and Super Protection). (2) - Paired balance games (e.g., statue game; no smiling; and do like me). - Work on imagery (e.g., a trip to your own planet “galaxy of feelings and behaviors”—imagine for 5 min; give emphasis to transmission to the others your sensations: smells, colors, details…). - And after in scenario paper, add all the planets to the “galaxy of feelings and behaviors” and give it an important role.
8	Promoting social–emotional competencies Main SEL domains: Self-knowledge Self-management Social consciousness Interpersonal relationship	(1) - “Intergalactic Friendship.” - “Emotions in Space.” - “My Superpowers”: What superpower would you like to receive? - Design it and give it creative name! - Delivery the Intergalactic Program Diploma. (2) - Drawing exercises and reflection on your “intergalactic friend” mediated by sharing in pairs and then in a large group about characteristics, behaviors and feelings associated with them. - Ventilation games and emotional expression (e.g., can “Intergalactics” express emotions just like us?), through movement and the body. Role-play exercises, freezes and puppets.

Based on a literature review (e.g., Sanders, 2008; Webster-Stratton, 2016), the program was designed as a psychoeducational and ludo-therapeutic resource. Each session has an average duration of 60 min, with a weekly frequency. The sessions incorporate relaxation dynamics and cognitive-behavioral training (e.g., Ferraioli and Harris, 2013; Black and Fernando, 2014; Raveepatarakul et al., 2014; Vickery and Dorjee, 2015; Huguet et al., 2017), as there is evidence showing that cognitive and behavioral interventions are a promising avenue for promoting social–emotional competencies, and self-control, in particular (Smith et al., 2019). Typically, the program is implemented by two trained group leaders, with one having a mandatory background in psychology.

A pilot study for this program was already conducted with 95 children, although with no control group (Antunes et al., 2022). The results showed that both younger children (8–9 years old) and older children (10–12 years old), regardless of gender, reported a reduction in psychopathological symptoms (anxiety, depression, and stress) and an improvement in overall socio-emotional skills from the pretest to the posttest and follow-up.

### The present study

Considering that multiple informants’ evaluations provide incremental validity beyond a single type of measurement (e.g., children’s self-report measures) and help capture differences in child behavior across different contexts (Alexander et al., 2017), and considering the limited number of studies conducted in Ibero-American regions on this topic, the present study aimed to evaluate the benefits of the “Intergalactic World” SEL program in Portugal from the perspectives of teachers and caregivers.

This study expected to observe a reduction in internalizing and externalizing behaviors from T1 to T2 and T3, with potential gender differences. Specifically, a significant reduction in externalizing behaviors was anticipated in the posttest and follow-up for boys, while girls were expected to exhibit lower overall levels of externalizing behaviors. For internalizing problems, based on the literature review, a significant reduction in internalizing behaviors was anticipated in the posttest and follow-up for girls, and it was expected that boys would exhibit lower overall levels of such behaviors.

By conducting research on SEL programs in diverse cultural and linguistic contexts, including Ibero-American regions, this study contributes to a better understanding of the efficacy and applicability of such programs across different populations. It also addresses the limited number of studies conducted in these regions, providing valuable insights into the potential benefits of the “Intergalactic World” program in Portugal.

## Methods

### Study design

The study employed a pretest-posttest design with a follow-up period and did not include a control group. The data were collected at three different time points: pre-intervention assessment (Time 1, T1), post-program evaluation (Time 2, T2), and 6-month follow-up (Time 3, T3). The T1 assessment was conducted from January to March 2019. Subsequently, the T2 assessment occurred in March and April, 1 week after the completion of the ‘Intergalactic World’ program. Finally, the T3 assessment was conducted in November and December, representing a follow-up evaluation conducted 6 months after the conclusion of the program, to examine the longer-term effects.

### Participants

A total of 154 children, aged between 8 and 12 years (*M* = 9.59 years, *SD* = 0.86), participated in this intervention study. All children attend public schools from the urban Lisbon area. Schools from areas of diverse socio-economic status were included. Eleven teachers provided information about these children. Almost all teachers were female (*n =* 10), with an average age of 42.25 years (*SD =* 4.62) and an average of 17.29 years of professional experience (*SD =* 4.15; *Min* = 11 years; *Max* = 21 years).

Caregivers provided information about 133 children. The majority of the caregivers were also female (*n =* 125) and had an average age of 41.14 years (*SD =* 6.63). Regarding the caregivers’ educational status, data showed that 30% of participating families were in the medium-qualified category, 22% were in the medium-qualified, and 49% were in the low-qualified category.^1^

Although initially 154 children participated in this intervention study, the final sample was limited to 133 children (*M* age = 9.66, *SD* = 0.78) due to the availability of the information collected from both parents and teachers. The final sample included a balanced distribution of boys and girls (52% boys and 48% girls).

### Measures

Caregivers completed a sociodemographic data questionnaire (i.e., age, gender, and academic qualifications) and the European Portuguese version of the Child Behavior Checklist (CBCL/6–18 version; Achenbach and Rescorla, 2000; Achenbach et al., 2014) at T1, T2, and T3 evaluation times. The CBCL is a 113-item standardized checklist administered to caregivers to detect behavioral and emotional problems in children and adolescents aged 6–18 years. Caregivers respond to the items on a three-point Likert scale (0—Not True, 1 — Somehow or Sometimes True, and 2—Very True or Often True). The time frame for item responses is the past 6 months. The CBCL includes syndrome scales combined to produce an Internalizing score (Anxious/Depressed, Withdrawn/Depressed, and Somatic Complaints) and an Externalizing score (Rule-Breaking Behavior and Aggressive Behavior). Higher scores in the CBCL indicate increased symptom severity, with T-scores above 69 indicating clinically elevated symptoms. For the Portuguese adaptation, Cronbach’s alphas for the dimensions of problems ranged between 0.845 (Internalization) and 0.957 (Total Problems) in the normative sample and between 0.846 (Internalization) and 0.934 (Total Problems) in a clinical sample. In the present sample, Cronbach’s alphas vary within the same range, and the average inter-item correlation for all the items on the internalization and externalization scales ranged between 0.66 and 0.72.

Teachers also completed a sociodemographic questionnaire (i.e., age, gender, and years of professional experience) and the European Portuguese version of the Teacher’s Report Form (TRF; Achenbach and Rescorla, 2000; Achenbach et al., 2014). The TRF is a questionnaire completed by teachers or other school personnel closely interacting with the child. From the teacher’s perspective, it assesses behavioral and emotional problems of children aged between 6 and 18 years. The TRF shares similarities with the CBCL regarding item content but is specifically adapted to capture behaviors and symptoms that are more relevant in the school environment. This tool consists of 113 items, answered on a three-point Likert scale (0—Not True, 1—Somehow or Sometimes True, and 2—Very True or Often True). The time frame for item responses is the past 2 months. The TRF includes syndrome scales combined to produce an Internalizing score (Anxiety/Depression, Withdrawn/Depressed, and Somatic Complaints) and an Externalization score (Rule-Breaking Behavior and Aggressive Behavior). Higher scores on the TRF also indicate greater symptom severity, with T-scores above 69 indicating clinically elevated symptoms. Test–retest reliabilities for the broadband scales range from 0.77 to 0.89, demonstrating acceptable consistency over time. For the Portuguese adaptation, Cronbach’s alphas ranged between 0.83 (Internalization) and 0.94 (Total Problems) in the normative sample and between 0.85 (Internalization) and 0.96 (Total Problems) in a clinical sample. In the current sample, Cronbach’s alphas in the present sample varied between 0.72 (Internalization at T2) and 0.97 (Total Problems and Externalization at T2), and the average inter-item correlation for all the items on both internalization and externalization scales ranged between 0.27 and 0.89.

### Procedures

This study was conducted as a part of a broader research project, approved by the Ethics Committee of one of the universities to which the authors belong and by the Survey Monitoring System from the Portuguese Education Ministry.

The “Intergalactic’s World” was presented to different schools during October and November 2018. Detailed contacts were made with the ones that expressed interest in implementing the program. Informed consent forms providing information about the study’s goals, the voluntary nature of participation, and the confidentiality of collected data were provided. Written authorization from the school boards was also obtained. After signing the informed consent, teachers completed the evaluation protocol. Before the beginning of the assessments, caregivers also provided written consent for their children’s participation in the study.

The intervention groups comprised approximately 20 children who met weekly for approximately 90 min per session. Each group was led by two trained group leaders who adhered to the structured manual and completed self-evaluations and checklists after each meeting to ensure program fidelity. Moreover, the group leaders received regular supervision. The participant’s compliance with the program was excellent (98% attendance rate), with only occasional absences due to illness. The attrition from intervention rate in this study was 2%.

The intervention sessions were conducted in school settings for most children. However, it is important to note that the intervention for 11 children occurred within a Social Solidarity Private Institution (IPSS), even though these children were also attending the same public schools.

The TRF questionnaire was completed with face-to-face support from the research team to clarify doubts and address additional questions. On the other hand, caregivers filled out the CBCL questionnaire at home. Both teachers and caregivers took an average of 2 weeks to answer the questionnaires at the three different time points. The research team provided contacts (such as email and meeting points) to clarify doubts and address additional questions.

### Data analysis

Data were analyzed using the Statistical Package for the Social Sciences (SPSS version 27). Distance-based outlier detection methods (i.e., Mahalanobis and Cook’s distances) revealed that the dataset did not include any outliers or influential points.

A repeated measures ANOVA was conducted to examine the effects of time (within-subjects variable) and gender (between-subjects variable) on Internalization and Externalization subscales of the CBCL and the TRF, as rated by caregivers and teachers. *Post hoc* analyses were conducted, and the effect sizes were reported using partial eta squared. Alpha was set at 0.05 for this model.

In this study, missing data were addressed using pairwise deletion. This method involves considering only the available data for each specific analysis. If a particular data point was missing, it was excluded only for that specific analysis, while the remaining existing data were used in the statistical testing. This approach helps to minimize biases and retain as much information as possible for the analysis.

As Mauchly’s test of sphericity yielded significant results, the Greenhouse–Geisser corrected *F* values were reported. In cases where a significant overall *F* value was observed, pairwise comparisons using Bonferroni correction were performed to compare the individual time points.

## Results

A Repeated Measures ANOVA was conducted to examine the impact of time (within-subjects) and gender (between-subjects) on the Internalization and Externalization scores obtained from the CBCL and TRF questionnaires. Table 2 provides a comprehensive overview of the means, standard deviations, and results of the repeated measures ANOVA for the Internalization and Externalization subscales.

**Table 2:** Outcomes’ descriptive statistics over time (T1, T2, and T3) and repeated measures ANOVA results.

Variables	Time (T)			Repeated measures ANOVA		Repeated measures ANOVA		
				Tests of within-subject effects		Pairwise Comparisons		
	T1	T2	T3	*F (df)*	*np2*	T1 vs. T2	T2 vs. T3	T1 vs. T3
	*M (SD)*	*M (SD)*	*M (SD)*					
Internalization subscale–Parents	54.08 (9.72)	51.19 (8.58)	40.23 (6.79)	369.93^*^ (1.199, 155.886)	0.74	3.01^*^	10.88^*^	13.89^*^
Internalization subscale—Teachers	46.21 (10.67)	46.82 (9.85)	39.10 (6.20)	43.849^*^ (1.790, 229.088)	0.26	−0.59	7.62^*^	7.03^*^
Externalization subscale—Parents	51.14 (9.32)	49.85 (8.02)	40.18 (7.88)	356.38^*^ (1.212, 157.580)	0.73	1.33^*^	9.57^*^	10.90^*^
Externalization subscale—Teachers	50.58 (10.80)	50.89 (10.8)	44.83 (6.40)	29.43^*^ (1.655, 211.860)	0.19	−0.26	6.10^*^	5.84^*^

T1, pre-intervention; T2, post-intervention; and T3, 6-month follow-up. ^*^p < 0.001.

The analysis of the CBCL data revealed a significant effect of time on the Internalization score, *F* (1.199, 155.886) = 369.929, *p* < 0.001. This indicated that time substantially influenced the Internalization score, with a moderate effect size (partial η^2^ = 0.74). It is important to note that the assumption of sphericity was violated, and Greenhouse–Geisser corrections were applied. The interaction effect between time and gender was not statistically significant (*F* < 1), suggesting that the relationship between time and the Internalization score did not differ based on gender. To further investigate the effects of time, within-subject contrasts were performed comparing the scores at T1, T2, and T3 on the Internalization scale. These comprehensive analyses thoroughly investigated the temporal dynamics and uncovered substantial differences across all time points, indicating a statistically significant score reduction at each assessed time point (*cf.* Table 2).

Regarding the Externalization score of CBCL, results also showed a significant effect of time, *F* (1.212, 157.580) = 356.379, *p* < 0.001. This finding indicated that time exerted a substantial influence on the Externalization score, yielding a moderate effect size (partial η^2^ = 0.73). It is worth noting that the assumption of sphericity was again violated, and the Greenhouse–Geisser corrections were used. Moreover, a statistically significant interaction effect between time and gender was observed, *F* (1, 130) = 6.986, *p* = 0.009, partial η^2^ = 0.51, suggesting that the relationship between time and the Externalization score differed depending on gender: overall, boys showed more externalizing behaviors than girls, but caregivers reported a reduction over time (*cf.* Table 2). In order to further examine the effects of time, within-subject contrasts were conducted, comparing the scores at T1, T2, and T3 on the Externalization scale. This analysis showed a statistically significant score reduction at each assessed time point (*cf.* Table 2).

The analysis of the TRF data revealed a statistically significant effect of time on the Internalization score as well, *F* (1.790, 229.088) = 43.849, *p* < 0.001. This indicated that time had a substantial impact on the Internalization score, with a moderate effect size (partial η^2^ = 0.26). The results showed that the relationship between time and the Internalization score did not differ based on gender, as the interaction effect was not statistically significant (*F* < 1). To further investigate the effects of time, within-subject contrasts were conducted, comparing the scores at T1, T2, and T3 on the Internalization scale. The within-subject contrasts revealed that only the scores at T3 showed a statistically significant reduction on the Internalization scale (*cf.* Table 2).

The analysis of the TRF data also demonstrated a significant effect of time on the Externalization score, *F* (1.655, 211.860) = 29.43, *p* < 0.001. This indicates that time substantially influenced the Externalization score, with a small effect size (partial η^2^ = 0.19). It is important to note that the assumption of sphericity was violated, and Greenhouse–Geisser corrections were applied. The interaction effect between time and gender was also not statistically significant (*F <* 1), suggesting that the relationship between time and the Internalization score did not differ based on gender. Within-subject contrasts performed comparing the scores at T1, T2, and T3 on the Externalization scale showed that only T3 presented a statistically significant score reduction (*cf.* Table 2).

## Discussion

Evidence-based SEL programs have been implemented worldwide. This study aimed to contribute to the evaluation of SEL programs in Portugal, specifically focusing on the “Intergalactic World” program developed for 8–12-year-old children (Cristóvão et al., 2017). Conducting research on social–emotional learning (SEL) programs in diverse cultural and linguistic contexts has enhanced our understanding of their efficacy and applicability across different populations.

The main objective of this study was to analyze the potential benefits of the “Intergalactic World” program from the perspectives of caregivers and teachers. Adopting a multi-informant approach allowed for a more comprehensive analysis and provided insights into the child’s significant contexts and interactions with adults (Alexander et al., 2017). This approach was particularly important when assessing new SEL programs as it helped provide evidence-based information and a more well-rounded perspective on their effectiveness.

Our results are in line with previous findings (Tennant et al., 2017; Scafuto et al., 2022), as teachers and caregivers reported reduced internalizing and externalizing behaviors in children who participated in SEL programs. This reduction was observed immediately after the intervention (T2) and remained evident 6 months after (T3).

However, our results differ from previous studies that have reported small-magnitude improvements in terms of externalizing problems, as perceived by teachers (e.g., Aber et al., 2003; Linares et al., 2005; Hennessey, 2007; Conduct Problems Prevention Research Group, 2010; Jones et al., 2011; Miller et al., 2017). One possible explanation for these differences is that our study focused on assessing short-term effects, as studies on the effectiveness of other SEL programs, such as PATHS in Switzerland (Malti et al., 2011) and 4R in the United States (Jones et al., 2011), which also observed changes in children’s aggressive behavior only in the long term (1–2 years after the end of the intervention).

Another potential factor contributing to the contrasting results could be the objectives and contents of the ‘Intergalactic’s World’ program intervention compared to other SEL interventions where teachers perceived short-term changes in externalizing problems. Specifically, school-age SEL interventions, such as Competent Kids, Caring Communities, RBI, RCCP, and Open Circle, lasted for more than 20 sessions and aimed not only to promote self-regulation skills but also to directly address communication skills, problem-solving, and positive peer relationships. On the other hand, the “Intergalactic’s World” program aimed to reduce internalizing, externalizing, and other problems (thinking, social, and attention) over eight sessions, incorporating dynamics of relaxation, mindfulness, and cognitive-behavioral training.

Despite its contribution, the study has some major limitations that should be addressed. Firstly, convenience sampling in the urban Lisbon area limits the generalizability of the findings to a broader population. Secondly, the absence of a control group raises the possibility that factors other than participation in the intervention may have influenced the observed results. Thus, the current study design only allows for partial assumptions on the program’s effects on internalizing and externalizing behaviors.

Moreover, the results found at T3 could be influenced by developmental trajectories, as suggested by previous research (Gutman and McMaster, 2020), and the instructions of the measures used by the present study. For instance, respondents were asked to fill in the CBCL and TRF questionnaires considering the last 2 or 6 months of the child’s life, which did not specifically target the end of the intervention but rather the entire intervention period.

Additionally, it is worth noting that between T2 and T3, the children had an extended vacation period due to the summer school break. This period off from school could have impacted the results, and it should be considered a potential confounding factor in the interpretation of the findings.

Suggestions for future studies include employing a randomized control trial design with a larger and more diverse sample from various regions of the country. These studies should also consider evaluating the long-term effectiveness of the intervention by conducting follow-ups 1–2 years after the end of the program.

Additionally, future research should adopt a multi-method approach, incorporating focus groups with children and group facilitators. These focus groups can provide valuable qualitative insights into program implementation and identify variables that may contribute to the program’s efficacy (Durlak et al., 2011).

Compelling evidence supports the effectiveness of interventions implemented in school contexts for reducing problem behaviors (e.g., Durlak et al., 2011; Taylor et al., 2017). Moreover, these interventions have positively impacted school achievement (Cristóvão et al., 2017). This study sheds light on the significant contributions of the “Intergalactic’s World” program, as perceived by teachers and caregivers, emphasizing the need for a stronger evidence-based approach to the program.

In summary, future research should apply more robust study designs, involve larger samples, and incorporate qualitative approaches to better understand the SEL program’s effectiveness and implementation. By doing so, we can further enhance the impact of SEL programs like the “Intergalactic’s World” on children’s well-being and development.

## Data availability statement

The original contributions presented in the study are included in the article/supplementary material, further inquiries can be directed to the corresponding authors.

## Ethics statement

The studies involving humans were approved by William James Center for Research—ISPA, Instituto Universitário, Portugal. The studies were conducted in accordance with the local legislation and institutional requirements. Written informed consent for participation in this study was provided by the participants’ legal guardians/next of kin. Written informed consent was obtained from the individual(s) for the publication of any potentially identifiable images or data included in this article.

## Author contributions

RA, MV, JA, and MG contributed to conception and design of the study. RA collected the data. RA and JA wrote the first draft of the manuscript. MF performed the statistical analysis, scoring, data entry, and writing the results. All authors contributed to the article and approved the submitted version.

## Funding

CIS_Iscte, Lisbon, Portugal, provided the funding for publishing; William James Center for Research—ISPA, Instituto Universitário, Lisbon, Portugal, and CIS_Iscte, Lisbon, Portugal, provided support for conducting the research (e.g., copies of materials for the program sessions, etc).

## Conflict of interest

The authors declare that the research was conducted in the absence of any commercial or financial relationships that could be construed as a potential conflict of interest.

## Publisher’s note

All claims expressed in this article are solely those of the authors and do not necessarily represent those of their affiliated organizations, or those of the publisher, the editors and the reviewers. Any product that may be evaluated in this article, or claim that may be made by its manufacturer, is not guaranteed or endorsed by the publisher.

## Footnotes

^1^Professional categories followed the employment/educational status classification of the Portuguese Classification of Jobs (retrieved from http://cdp.portodigital.pt/profissoes/classificacao-nacional-das-profissoes-cnp).

## References

[ref1] Aber J. L. Brown J. L. Jones S. M. (2003). Developmental trajectories toward violence in middle childhood: Course, demographic differences, and response to school-based intervention. Dev. Psychol. 39, 324–348. doi: 10.1037/0012-1649.39.2.324, PMID: 12661889

[ref2] Achenbach T. M. Edelbrock C. S. (1978). The classification of child psychopathology: a review and analysis of empirical efforts. Psychol. Bull. 85, 1275–1301. doi: 10.1037/0033-2909.85.6.1275, PMID: 366649

[ref3] Achenbach T. M. Rescorla L. A. (2000). Manual for the ASEBA School-Age Forms and Profiles University of Vermont, Research Center for Children, Youth, and Families.

[ref4] Achenbach T. M. Rescorla L. A. Dias P. Ramalho V. Lima V. S. Machado B. C.. (2014). Manual do Sistema de Avaliação Empiricamente Validado (ASEBA) Para o Período Pré-Escolar e Escolar. Psiquilíbrios Edições: Um sistema integrado de avaliação com múltiplos informadores.

[ref5] Alexander L. A. McKnight P. E. Disabato D. J. Kashdan T. B. (2017). When and how to use multiple informants to improve clinical assessments. J. Psychopathol. Behav. Assess. 39, 669–679. doi: 10.1007/s10862-017-9607-9, PMID: 37553655

[ref6] Antunes R. (in press). Programa de Competências Socioemocionais: “O Mundo dos Intergalácticos”. Lisboa: Ideias com História.

[ref7] Antunes R. Guedes M. Alexandre J. Veríssimo M. (2022). Benefícios de um novo programa de aprendizagem socioemocional na redução da sintomatologia psicopatológica e na promoção das competências socioemocionais globais, na perspetiva das crianças. Psicologia 36, 119–135. doi: 10.17575/psicologia.1813

[ref8] Black D. S. Fernando R. (2014). Mindfulness training and classroom behavior among lower-income and ethnic minority elementary school training. J. Child Fam. Stud. 23, 1242–1246. doi: 10.1007/s10826-013-9784-4, PMID: 25624749 PMC4304073

[ref32] Chatterjee Singh N. Duraiappah A. K. (Eds.). (2020). Rethinking learning: a review of social and emotional learning frameworks for education systems. New Delhi: UNESCO MGIEP.

[ref9] Chernyshenko O. Kankaras M. Drasgow F. (2018). Social and Emotional Skills for Student Success and Well-Being: Conceptual Framework for the OECD Study on Social and Emotional Skills. OECD Education Working Papers, No. 173. Paris: OECD Publishing.

[ref10] Collaborative for Academic, Social, and Emotional Learning (2012). 2013 CASEL Guide: Effective Social and Emotional Learning Programs—Preschool and Elementary School Edition. Chicago, IL: Author.

[ref11] Conduct Problems Prevention Research Group (2010). The effects of a multiyear universal social-emotional learning program: The role of student and school characteristics. J. Consult. Clin. Psychol. 78, 156–168. doi: 10.1037/a0018607, PMID: 20350027 PMC3534742

[ref12] Cristóvão A. M. Candeis A. A. Verdasca J. (2017). Social and emotional learning and academic achievement in Portuguese schools: A bibliometric study. Front. Psychol. 8:1913. doi: 10.3389/fpsyg.2017.01913 29167650 PMC5682338

[ref13] Durlak J. A. Weissberg R. P. Dymnicki A. B. Taylor R. D. Schellinger K. B. (2011). The impact of enhancing students’ social and emotional learning: A meta-analysis of school-based universal interventions. Child Dev. 82, 405–432. doi: 10.1111/j.1467-8624.2010.01564.x, PMID: 21291449

[ref14] Eme R. (2016). “Sex differences in the prevalence and expression of externalizing behavior” in The Oxford Handbook of Externalizing Spectrum Disorders. eds. Beauchaine T. P. Hinshaw S. P. (Oxford University Press), 239–263.

[ref15] Fernández-Martín F.-D. Romero-Rodríguez J.-M. Marín-Marín J.-A. Gómez-García G. (2021). Social and emotional learning in the Ibero-American context: A systematic review. Front. Psychol. 12:738501. doi: 10.3389/fpsyg.2021.738501 34659053 PMC8516388

[ref16] Ferraioli S. J. Harris S. L. (2013). Comparative effects of mindfulness and skills-based parent training programs for caregivers of children with autism: Feasibility and preliminary outcome data. Mindfulness 4, 89–101. doi: 10.1007/s12671-012-0099-0

[ref17] Gutman L. M. McMaster N. (2020). Gendered pathways of internalizing problems from early childhood to adolescence and associated adolescent outcomes. J. Abnorm. Child Psychol. 48, 703–718. doi: 10.1007/s10802-020-00623-w, PMID: 32040796 PMC7188729

[ref18] Hansen L. K. Jordan S. S. (2020). “Internalizing behaviors” in Encyclopedia of Personality and Individual Differences. eds. Zeigler-Hill V. Shackelford T. K. (Springer International Publishing), 2343–2346.

[ref19] Hennessey B. A. (2007). Promoting social competence in school-aged children: The effects of the Open Circle Program. J. Sch. Psychol. 45, 349–360. doi: 10.1016/j.jsp.2006.11.007

[ref20] Huguet A. Ruiz D. M. Haro J. M. Alda J. A. (2017). A pilot study of the efficacy of a mindfulness program for children newly diagnosed with attention-deficit hyperactivity disorder: Impact on core symptoms and executive functions. Int. J. Psychol. Psychol. Ther. 17, 305–316.

[ref21] Jones S. M. Brown J. L. Aber J. L. (2011). Two-year impacts of a universal school-based social-emotional learning and literacy intervention: An experiment in translational developmental research. Child Dev. 82, 533–554. doi: 10.1111/j.1467-8624.2010.01560.x, PMID: 21410922

[ref22] Kankaraš M. Suarez-Alvarez J. (2019). Assessment framework of the OECD study on social and emotional skills. OECD Educ. Work. Papers 207. doi: 10.1787/5007adef-en

[ref23] Lau T. W. I. Lim C. G. Acharryya S. Lim-Ashworth N. Tan Y. R. Fung S. S. D. (2021). Gender differences in externalizing and internalizing problems in Singaporean children and adolescents with attention-deficit/hyperactivity disorder. Child Adolesc. Psychiatry Ment. Health 15:3. doi: 10.1186/s13034-021-00356-8 33482840 PMC7825195

[ref24] Linares L. O. Rosbruch N. Stern M. B. Edwards M. E. Walker G. Abikoff H. B.. (2005). Developing cognitive-social-emotional competencies to enhance academic learning. Psychol. Sch. 42, 405–417. doi: 10.1002/pits.20066

[ref25] Malti T. Ribeaud D. Eisner M. P. (2011). The effectiveness of two universal preventive interventions in reducing children’s externalizing behaviour: a cluster randomized controlled trial. J. Clin. Child Adolesc. Psychol. 40, 677–692. doi: 10.1080/15374416.2011.597084, PMID: 21916687

[ref26] Miller C. F. Kochel K. P. Wheeler L. A. Updegraff K. A. Fabes R. A. Martin C. L.. (2017). The efficacy of a relationship building intervention in 5th grade. J. Sch. Psychol. 61, 75–88. doi: 10.1016/j.jsp.2017.01.002, PMID: 28259245

[ref27] Raveepatarakul J. Suttiwan P. Iamsupasit S. Mikulas W. L. (2014). A mindfulness enhancement program for Thai 8- to 11-year old children: effects on mindfulness and depression. J. Health Res. 28, 335–341

[ref28] Sanders M. R. (2008). Triple P-Positive Parenting Program as a public health approach to strengthening parenting. J. Fam. Psychol. 22, 506–517. doi: 10.1037/0893-3200.22.3.506 18729665

[ref29] Scafuto F. Ghiroldi S. Montecucco P. F. Iani L. (2022). The Mindfulness-Based Gaia Program reduces internalizing problems in high-school adolescents: a cluster randomized controlled trial. Mindfulness 13, 1804–1815. doi: 10.1007/s12671-022-01920-9

[ref30] Schuman-Olivier Z. Trombka M. Lovas D. A. Brewer J. A. Vago D. R. Gawande R.. (2020). Mindfulness and Behavior Change. Harv. Rev. Psychiatry 28, 371–394. doi: 10.1097/HRP.0000000000000277, PMID: 33156156 PMC7647439

[ref31] Scott S. Knapp M. Henderson J. Maughan B. (2001). Financial cost of social exclusion: follow up study of antisocial children into adulthood. BMJ 323, 191–194. doi: 10.1136/bmj.323.7306.191, PMID: 11473907 PMC35269

[ref33] Smith T. Panfil K. Bailey C. Kirkpatrick K. (2019). Cognitive and behavioral training interventions to promote self-control. J. Expe. Psychol. Anim. Learn. Cogn. 45, 259–279. doi: 10.1037/xan0000208, PMID: 31070430 PMC6716382

[ref34] Taylor R. D. Oberle E. Durlak J. A. Weissberg R. P. (2017). Promoting positive youth development through school-based social and emotional learning interventions: A meta-analysis of follow-up effects. Child Dev. 88, 1156–1171. doi: 10.1111/cdev.12864, PMID: 28685826

[ref35] Tennant R. G. Martin K. K. Rooney R. Hassan S. Kane R. T. (2017). Preventing internalizing problems in young children: A randomized controlled trial of the feelings and friends (Year 3) program with a motor skills component. Front. Psychol. 8:291. doi: 10.3389/fpsyg.2017.00291, PMID: 28326047 PMC5339246

[ref36] Vickery C. Dorjee D. (2015). Mindfulness training in primary schools decreases negative affect and increases meta-cognition in children. Front. Psychol. 6:2025. doi: 10.3389/fpsyg.2015.02025 26793145 PMC4709470

[ref37] Webster-Stratton C. (2016). “The Incredible Years® series: a developmental approach,” in Family-Based Prevention Programs for children and Adolescents: Theory, Research, and Large-Scale Dissemination. (eds.) Ryzin M. J. Van Kumpfer K. L. Fosco G. M. Greenberg M. T. (New York: Psychology Press), 42–67

